# Supplementary material for: A simpler screening tool for sarcopenia in surgical patients
Source: PLoS One. 2021 Sep 23;16(9):e0257672. doi: 10.1371/journal.pone.0257672 (PMC8460047; doi:10.1371/journal.pone.0257672)
Supplement: Attachment — Submitted filename: Response to reviewer__5092021.pdf [file pone.0257672.s007.pdf]

Manuscript number: PONE-D-21-00876R1
Journal: PLOS ONE
Title: A simpler screening tool for sarcopenia in surgical patients

Journal Requirements:

Please review your reference list to ensure that it is complete and correct. If you have cited papers that have been retracted, please include the rationale for doing so in the manuscript text, or remove these references and replace them with relevant current references. Any changes to the reference list should be mentioned in the rebuttal letter that accompanies your revised manuscript. If you need to cite a retracted article, indicate the article's retracted status in the References list and also include a citation and full reference for the retraction notice.

Response: We reviewed and explained the retracted references and the added references in the tables below accompanied with the revised manuscript.

**Removed References**	**Reasons**
Chow WB, Rosenthal RA, Merkow RP, Ko CY, Esnaola NF. Optimal preoperative assessment of the geriatric surgical patient: a best practices guideline from the American College of Surgeons National Surgical Quality Improvement Program and the American Geriatrics Society. J Am Coll Surg. 2012;215(4):453-66.	We removed this sentence and one reference to shorten the background. (Background)<br><br>*"Recent guidelines from the American College of Surgeons emphasize the importance of assessing sarcopenia prior to oncologic surgery in elderly patients."*
Han A, Bokshan SL, Marcaccio SE, DePasse JM, Daniels AH. Diagnostic Criteria and Clinical Outcomes in Sarcopenia Research: A Literature Review. J Clin Med. 2018;7(4).	We removed these sentences and one reference to shorten the discussion. (Discussion)<br><br>*"Although the EWGSOP has published sarcopenia diagnostic criteria since 2010, many studies defined and reported the prevalence of sarcopenia from muscle mass measurement criteria only. Methods of measuring muscle mass included dual X–ray absorptiometry (DEXA), BIA, and total psoas area/volume or third lumbar (L3) vertebra muscle index derived from computerized tomography (CT) scan."*
Chakedis J, Spolverato G, Beal EW, Woelfel I, Bagante F, Merath K, et al. Pre-operative Sarcopenia Identifies Patients at Risk for Poor Survival After Resection of Biliary Tract Cancers. J Gastrointest Surg. 2018;22(10):1697-708.	We removed this sentence and one reference to shorten the discussion. (Discussion)<br><br>*"The prevalence of sarcopenia ranged from 12–47% in oncology gastrointestinal surgical patients."*
Zhou CJ, Zhang FM, Zhang FY, Yu Z, Chen XL, Shen X, et al. Sarcopenia: a new predictor of postoperative complications for elderly gastric cancer patients who underwent radical gastrectomy. J Surg Res. 2017;211:137-46.	We removed these sentences and one reference to shorten the discussion. (Discussion)<br><br>*"Previous study from China in oncology surgical patients defined sarcopenia by the combination of*

	low muscle mass and/or low muscle strength and low physical performance. Zhou C, et al. reported a prevalence of sarcopenia of 28.8% in gastric cancer patients who underwent radical gastrectomy among a cohort of 240 patients."
Huang DD, Wang SL, Zhuang CL, Zheng BS, Lu JX, Chen FF, et al. Sarcopenia, as defined by low muscle mass, strength and physical performance, predicts complications after surgery for colorectal cancer. Colorectal Dis. 2015;17(11):O256-64.	We removed these sentences and one reference to shorten the discussion. (Discussion)<br><br>*"Those researchers used AWGS cutoff values for grip strength and gait speed, but they used a cross-sectional CT image of muscle at the inferior aspect of L3 to estimate muscle mass. Another study from the same research group in colorectal cancer patients used the same criteria to diagnose sarcopenia, and the prevalence of sarcopenia was found to be only 12% among 142 patients."*
Janssen I, Heymsfield SB, Baumgartner RN, Ross R. Estimation of skeletal muscle mass by bioelectrical impedance analysis. J Appl Physiol (1985). 2000;89(2):465-71.	We removed these sentences and one reference to shorten the discussion. (Discussion)<br><br>*"Although the accuracy of BIA is dependent on many conditions (e.g., temperature, humidity, and the accuracy of the equation of the equipment), it is convenient, portable, low cost, and there is no radiation exposure. Taken together, these findings indicate a range of prevalence rates that vary by race, type of cancer, study design, type of surgery, and the criteria used to diagnose sarcopenia."*
Senior HE, Henwood TR, Beller EM, Mitchell GK, Keogh JW. Prevalence and risk factors of sarcopenia among adults living in nursing homes. Maturitas. 2015;82(4):418-23.	We removed this sentence and one reference to shorten the discussion. (Discussion)<br><br>*"Senior, et al. reported only BMI to be related to sarcopenia in nursing home population."*

**Added References no.**	**Reasons**
14. Chen LK, Woo J, Assantachai P, Auyeung TW, Chou MY, Iijima K, et al. Asian Working Group for Sarcopenia: 2019 Consensus Update on Sarcopenia Diagnosis and Treatment. J Am 452 Med Dir Assoc. 2020;21:300-7.e2.	We added this sentence as recommended by the reviewer. (Background, Page 3, Line 68-71)<br><br>*"The recently updated 2019 AWGS consensus contains revisions to the diagnostic algorithm, the protocols, and some criteria, including the cut-off value for low muscle strength and low physical performance. Nevertheless, skeletal muscle strength and mass remain foundational to a definitive clinical diagnosis of sarcopenia. [14]"*
16. Yilmaz O, Bahat G. Suggestions for assessment of muscle mass in primary care setting. Aging Male. 2017;20:168-9.	We added this sentence and one reference as recommended by the reviewer. (Background, Page 3-4, Line 74-75)<br><br>*"BIA is practical, and portable application that does not expose the patient to any radiological harms.[16]"*

25. Bahat G, Oren MM, Yilmaz O, Kılıç C, Aydin K, Karan MA. Comparing SARC-F with SARC-CalF to Screen Sarcopenia in Community Living Older Adults. J Nutr Health Aging. 478 2018;22:1034-8.<br>26. Barbosa-Silva TG, Menezes AM, Bielemann RM, Malmstrom TK, Gonzalez MC. Enhancing SARC-F: Improving Sarcopenia Screening in the Clinical Practice. J Am Med Dir Assoc. 481 2016;17:1136-41.	We added this sentence and 2 references as recommended by the reviewer. (Background, Page 4, Line 89-90)<br><br>*"Calf circumference was incorporated in SARc-CalF as an additional parameter to enable indirect measuring of muscle mass.[25, 26]"*
35. Bahat G, İlhan B. Sarcopenia and the cardiometabolic syndrome: A narrative review. Eur Geriatr Med. 2016;7:220-3.	We added these sentences and one reference as recommended by the reviewer. (Discussion, Page 15-16, Line 310-314)<br><br>*"In addition, we demonstrated that the sarcopenic group had a significantly lower number of patients with DM and DLP. Hypothetically, sarcopenia should be related to higher DM and dyslipidemia due to the loss of metabolically active muscle tissue. However, sarcopenia was reported to be associated with lower DM if muscle mass was adjusted by height2, but it was associated with a higher DM if the muscle mass was adjusted by weight or BMI.[35]"*
43. Kyle UG, Bosaeus I, De Lorenzo AD, Deurenberg P, Elia M, Gomez JM, et al. Bio- electrical impedance analysis—part I: review of principles and methods. Clin Nutr 2004;23:1226–43	We added this sentence and one reference as recommended by the reviewer. (Discussion, Page 18, Line 376-378)<br><br>*"Thirdly, the fat-free mass and body cell mass measured by BIA were calculated from the total body weight, using the assumption that 73% of the fat-free mass was water. Therefore, changes in the hydration state, such as edema, were the main limitation of this method.[43]"*

<u>Additional Editor Comments</u> (if provided):

The study has many limitations which are addressed in this new version. I also consider as an important concern the inclusion of patients with oedema, so the authors should mention it in the limitation section.

Response: We added this limitation as recommended.

"Thirdly, the fat-free mass and body cell mass measured by BIA were calculated from the total body weight, using the assumption that 73% of the fat-free mass was water. Therefore, changes in the hydration state, such as edema, were the main limitation of this method." (Discussion, Page 18, Line 376-378)

Reviewers' comments:

Reviewer's Responses to Questions

Comments to the Author

1. If the authors have adequately addressed your comments raised in a previous round of review and you feel that this manuscript is now acceptable for publication, you may indicate that here to bypass the "Comments to the Author" section, enter your conflict of interest statement in the "Confidential to Editor" section, and submit your "Accept" recommendation.

Reviewer #2: All comments have been addressed
Reviewer #3: (No Response)

2. Is the manuscript technically sound, and do the data support the conclusions?

The manuscript must describe a technically sound piece of scientific research with data that supports the conclusions. Experiments must have been conducted rigorously, with appropriate controls, replication, and sample sizes. The conclusions must be drawn appropriately based on the data presented.

Reviewer #2: Yes
Reviewer #3: Yes

3. Has the statistical analysis been performed appropriately and rigorously?

Reviewer #2: Yes
Reviewer #3: Yes

4. Have the authors made all data underlying the findings in their manuscript fully available?

The PLOS Data policy requires authors to make all data underlying the findings described in their manuscript fully available without restriction, with rare exception (please refer to the Data Availability Statement in the manuscript PDF file). The data should be provided as part of the manuscript or its supporting information, or deposited to a public repository. For example, in addition to summary statistics, the data points behind means, medians and variance measures should be available. If there are restrictions on publicly sharing data—e.g. participant privacy or use of data from a third party—those must be specified.

Reviewer #2: Yes
Reviewer #3: Yes

5. Is the manuscript presented in an intelligible fashion and written in standard English?

PLOS ONE does not copyedit accepted manuscripts, so the language in submitted articles must be clear, correct, and unambiguous. Any typographical or grammatical errors should be corrected at revision, so please note any specific errors here.

Reviewer #2: Yes
Reviewer #3: No

6. Review Comments to the Author

Please use the space provided to explain your answers to the questions above. You may also include additional comments for the author, including concerns about dual publication, research ethics, or publication ethics. (Please upload your review as an attachment if it exceeds 20,000 characters)

Reviewer #2: Most of my concerns are addressed, but the text still needs a minör revision:

1.Please prefer the term "older adults" instead of "elderly" throughout the text

Response: We corrected as recommended (highlighted in the manuscript)

2.The text still needs a rigorous editing,

Response: We resent for the English editing.

3.My suggestions for some phrases that cause misunderstanding are as follows:

Introduction

Line 77-78: The sentence'' the DXA and BIA have some limitation in terms of the accessibility, and costly equipment''. Please add the plural suffix ''s'' to the word limitation. Instead of ''costly equipman'' please write ''cost'' only.

Response: We corrected as recommended.

"*Both devices have some limitations in terms of their accessibility and cost*" (Background, Page 4, Line 76-77)

Line 95: In the sentence …….any one tool……, please remove the word ''one'', write as ….any tool…

Response: We corrected as recommended. (Background, Page 4, Line 92)

Line 97: Instead of ʹʹdiagnosis performanceʹʹ, please write ʹʹdiagnostic performanceʹʹ

Response: We changed to " *to validate its performance in screening for sarcopenia*" . (Background, Page 4, Line 94-95)

Material and methods

Line 128: Other collected data included……Here the term other refers to components of MNA-SF tool. But it is perceived as a different data apart from MNA-SF tool. I recommend combine with previous sentence and you may write as ʻʻ Preoperative nutritional screening was performed using the Mini Nutritional Assessment - Short Form (MNA® 128 –SF), which included reduction in dietary intake within the past three months, body mass index (BMI), history of weight loss within the last three months, mobility, psychological stress and/ or acute disease within the past 3 months, and neuropsychological problems.ʹʹ

Response: We corrected as recommended. (Materials and methods, Page 6, Line 121-124)

Results

Line 249: Please rewrite subheading, you may write as ʹʹ Diagnostic performance of a simple tool for screening sarcopeniaʹʹ

Response: After English editing, we corrected to "*Screening performance of the tool*". (Results, Page 12, Line 238)

Line 250-252: Please rewrite the sentence ʺThe combination of low muscle strength and risk of malnutrition & malnutrition and/ or abnormal physical performance (C3) showed the highest sensitivity and accuracy either the AWGS or the updated AWGS as the gold standardsʹʹ you may write as ʻʻ The combination of low muscle strength and risk of malnutrition & malnutrition and/ or abnormal physical performance (C3) showed the highest sensitivity and accuracy when using the AWGS or the updated AWGS as the gold standardsʹʹ

Response: We corrected as recommended**.**

" *Formula- combination C3 (low muscle strength and/or abnormal physical performance, plus malnutrition/risk of malnutrition) demonstrated the highest sensitivity and accuracy when using the 2014 AWGS or the updated 2019 AWGS criteria as the gold standards*." (Results, Page 12, Line 239-241)

Line 252-254: Please rewrite the sentence ʹʹ The sensitivity, specificity, accuracy and AUROC were 81.0%, 78.4%, 79.3% and 0.8, respectively as compared to C1, C2 and C4 when using AWGS as a gold standardʹʹ. In this sentence percent results are belong to C3, but C3 is not mentioned in the sentence, in addition sentence gives the statistical information only related to C3, there is not a comparison between the statistical results of the formulas. You may rewrite the sentence as ʹʹ The sensitivity, specificity, accuracy and AUROC of C3 were 81.0%, 78.4%, 79.3% and 0.8, respectively when using AWGS as gold standard.ʹʹ

Response: We corrected as recommended.

"The sensitivity, specificity, accuracy, and AUROC of formula-combination C3 were 81.0%, 78.4%, 79.3%, and 0.8, respectively when using the 2014 AWGS criteria as the gold standard." (Results, Page 12, Line 241-243)

Line 264-266: with similar reasons as above mentioned you may rewrite the sentence as '' The sensitivity, specificity, accuracy, and AUROC of C3 were 80%, 68. 5%, 73. 3% and 0.74 respectively when using the updated AWGS criteria as gold standard (Table 4b).''

Response: We corrected as recommended.

"The sensitivity, specificity, accuracy, and AUROC of formula C3 was 80%, 68.5%, 73.3%, and 0.74, respectively, when using the updated 2019 AWGS criteria as the gold standard (Table 4b)" (Results, Page 13, Line 253-255)

Discussion

Line 306-308: Please rewrite the sentence, you may rewrite as '' The combination of low muscle strength and risk of malnutrition & malnutrition and/ or abnormal physical performance (C3), demonstrated high sensitivity, specificity, and predictive power when validated against a consensus of the Asian Working Group for Sarcopenia (AWGS).

Response: We corrected as recommended.

"The combination of low muscle strength and/or abnormal physical performance, plus malnutrition/ risk of malnutrition (formula- combination C3), demonstrated high sensitivity, specificity, and predictive power when validated against a consensus of the AWGS." (Discussion, Page 15, Line 295-298)

Line 329: Please write immobile instead of immobilization

Response: After English editing, we corrected as recommended by the English editor.

" However, these tools are expensive, difficult to access, and not portable." (Discussion, Page 16, Line 319-320)

Reviewer #3: *English should be improved in the whole writing.

Abstract

***Page 2, Line 27:** 'Non-portable' should be used instead of immobile device.

Response: We corrected as recommended by the English editor.

" However, the devices used for these methods are costly and are usually not portable." (Abstract, Page 2, Line 26-27)

***Page 2, Line 28:** The aim of this study was to design and validate the diagnostic performance of a simple screening tool for screening sarcopenia.

These formula are not suggested for diagnosis of sarcopenia. Therefore, 'diagnostic' term should not be used.

Response: We changed "diagnostic" to "screening" performance.

***Page 2, Line 32:** The details about this screening tool should be explained in methods part.

Which parameters were considered for analyses, the details about formula should be briefly mentioned.

Response: We added as recommended.

"Four screening formulas with differing combinations of factors (muscle strength, physical performance, and nutritional status) were assessed. The validities of the formulas, compared with the AWGS definition, are presented as sensitivity, specificity, accuracy, and area under a receiver operating characteristic curve." (Abstract, Page 2, Line 34-37)

*** Page 2, Line 37:** Malnutrition and underweight status are surely expected to be strongly related.

Therefore, before putting into the same regression analysis, multicollinearity should be checked.

Response: The multicollinearity test was checked and demonstrated in tables below. The p-value for the F-test was less than 0.05 and the coefficients output collinearity statistics obtained variance inflation factor (VIF) value of 1.081. As a result, there was no multicollinearity.

ANOVA[a]

Model		Sum of Squares	df	Mean Square	F	Sig.
1	Regression	13.906	4	3.477	20.372	.000[b]
	Residual	41.982	246	.171		
	Total	55.888	250			

a. Dependent Variable: Sarcopenia

b. Predictors: (Constant), Pre–Barthel Index score < 70, Body mass index, Age (years, 5 units), Malnutrition

Coefficientsa

Model		Unstandardized Coefficients		Standardized Coefficients	t	Sig.	Collinearity Statistics	
		B	Std. Error	Beta			Tolerance	VIF
1	(Constant)	-1.085	.258		-4.209	.000		
	Age (years, 5 units)	.095	.018	.303	5.372	.000	.962	1.039
	Underweight	-.090	.030	-.174	-3.037	.003	.925	1.081
	Malnutrition	.214	.055	.222	3.896	.000	.937	1.067
	Pre–Barthel Index score < 70	.410	.171	.133	2.393	.017	.991	1.009

a. Dependent Variable: Sarcopenia

Background

***Page 3, Line 57:** In whole writing, elderly term should be changed to older adults.

Response: We changed as recommended.

***Page 3, Line 62:** Muscle function can be divided into those that require both muscle strength and physical functionality or only one of these elements.

Functionality should be revised as 'performance'.

Response: We changed as recommended.

*Introduction should be shortened.

Response: We shortened the introduction as recommended.

Materials and methods

***Page 5,** Study population exclusion criteria: Edema can also affect the BIA results, therefore, measurements without regarding edema status should also be stated as a limitation of the study.

Response: We added as recommended.

"Thirdly, the fat-free mass and body cell mass measured by BIA were calculated from the total body weight, using the assumption that 73% of the fat-free mass was water. Therefore, changes in the hydration state, such as edema, were the main limitation of this method." (Discussion, Page 18, Line 376-378)

***Page 7, Line 155:** At risk and malnutrition and underweight BMI were considered to be factors for diagnosing sarcopenia.

Underweight BMI should be changed as 'Being underweight by BMI'. Again, language revision should be done seriously.

Response: We corrected as recommended.

Results

*** Page 12, Line 249**: Diagnosis performance a simple tool for screening sarcopenia Diagnostic performance term should not be used.

These formula were suggested for sarcopenia screening, so 'screening' and 'diagnosis' terms should not be used interchangeably.

Response: We changed to "*Screening performance of the tool"***.** (Results, Page 12, Line 238)

***Page 13, Line 256:** EWGSOP2 demonstrated the highest specificity (100%).

Whether the high specificity proposed by EWGSOP2 is for probable sarcopenia or confirmed sarcopenia should be stated.

Response: We add "for diagnosis of sarcopenia" after "EWGSOP2 demonstrated the highest specificity (100%)". (Results, Page 12, Line 245)

Discussion

***Page 18, Line 355:** Although, there was a slightly low specificity and PPV in female, we introduced a practical algorithm for a diagnosis of sarcopenia in elderly cancerous surgical patients without using muscle mass measurement (Fig 2).

The algorhythm the authors proposed for sarcopenia diagnosis seems problematic. The authors should decide whether they propose this formula for screening (finding cases) or assessment of sarcopenia. In addition, muscle mass measurement was included for diagnosis in the algorhythm. But they claimed that the algorhythm enables to diagnose sarcopenia without muscle mass measurement. There seems a paradox.

Response: We changed the fig. 2 as shown below.

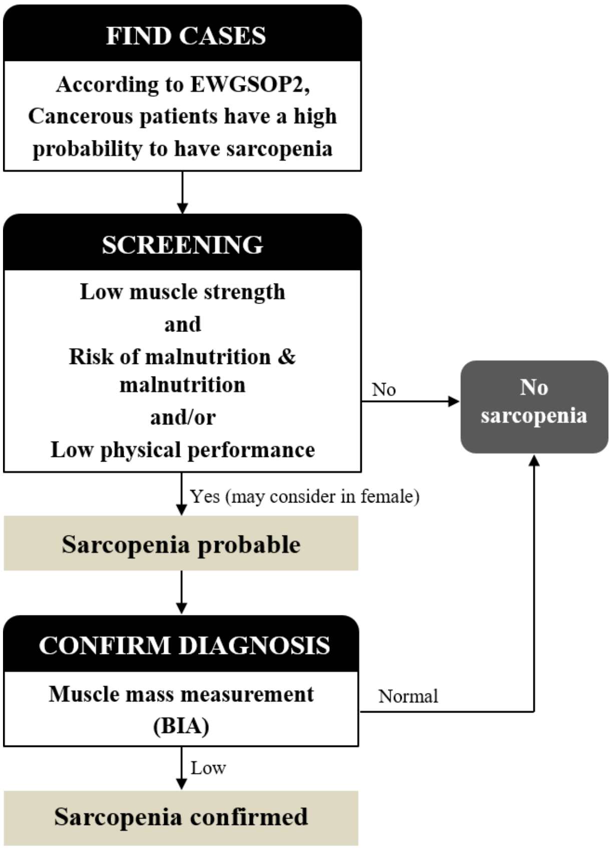

Conclusion

* Conclusion should be shortened. The first two sentences are the findings previously mentioned, unnecessary to repeat at the end.

Response: We changed as recommended. (Conclusion, Page 19, Line 390-396)

7. PLOS authors have the option to publish the peer review history of their article (what does this mean?). If published, this will include your full peer review and any attached files.

If you choose “no”, your identity will remain anonymous but your review may still be made public.

Do you want your identity to be public for this peer review? For information about this choice, including consent withdrawal, please see our Privacy Policy.

Reviewer #2: Yes: Firuzan Fırat Özer
Reviewer #3: Yes: Gulistan Bahat
