# Supplementary material for: A simpler screening tool for sarcopenia in surgical patients
Source: PLoS One. 2021 Sep 23;16(9):e0257672. doi: 10.1371/journal.pone.0257672 (PMC8460047; doi:10.1371/journal.pone.0257672)
Supplement: Attachment — Submitted filename: Review.docx [file pone.0257672.s002.docx]

I read the text with great interest. Authors explored the operational definition of sarcopenia according to AWGS criteria in a vulnerable patient population. % 34 of elderly cancer patients had sarcopenia and the sarcopenia in these patient population was significantly related with poor outcomes. Authors introduced a simpler algorithm for predicting sarcopenia by excluding the muscle mass measurement and incorporating MNA-SF tool. Presence of cachexia and secondary sarcopenia in cancer patients emphasized the importance of nutritional screening.

It is a prospective, well organized study and authors may consider the following comments:

1. The authors named the simpler method of defining sarcopenia, as a validated tool. However I think it will be better to present this as a simle algorithm not a validated tool. Actually authors are defining a simpler form of algorithm by incorporating MNA-SF instead of muscle mass into the operational definition of sarcopenia. I recommend to change as a simple algorithm instead of a validated tool in the title and in the text.
2. AWGS has updated consensus in 2019 and changed the cut-off values for handgrip strength for men (<28kg) and also changed cut-off criteria for low physical performance for 6-m walk (<1.0 m/s). In the text, the old AWGS cut off points reported in 2014 were used. I wonder why authors did not use the cut-offs defined in updated AWGS.
3. In table 1 surgical oncologic patients with sarcopenia had poorer outcomes such as lower Barthel index score at 3 months of discharge, higher mortality rate at 3 months and 1 year after discharge again. These outcome findings are valuable and have scientific impact. But these outcomes are not mentioned in result section and not discussed in discussion section. I recommend to highlight these outcomes both in result and discussion sections.
4. I also wonder if sarcopenia, when defined according to your new algorithm, is related with poor outcome measures?
5. Authors defining a flowchart for screening of sarcopenia in Figure 1. Figure 1 represents the sarcopenia screening algorithm of EWGSOP in 2010 by starting with gait speed only. Unlike EWGSOP, AWGS recommends measuring both muscle strength (handgrip strength) and physical performance (usual gait speed) as the screening test. Actually the final result does not change, but still the authors can rearrange figure 1 according to the AWGS algorithm.

Minor points

Abstract

1. Line 26: muscle mass and muscle functions (strength and function). Please use physical performance term instead of function in parentheses.
2. Line 27: Please change the word immobilization with immobile

Result section

1 Line 171-173: Please rewrite the sentence, it is not clear ‘’ Of those, only 84 subjects demonstrated low muscle mass below the recommended cutoff value for a prevalence of sarcopenia in this cohort of 34% .’’
3. Line 175-176: Presarcopenia was found in another 34 patients (40% ); however, these patients were included in the non–sarcopenia group for all other analyses’’. Here the term presarcopenia refers to patients only with low muscle mass not accompanying low muscle strength and muscle performance. I couldn't catch the rate 40% . 34 out of 104 patients have low muscle mass ?? In addition the term presarcopenia was included in EWGSOP in 2010, not addressed in revised EWGSOP, AWGS in 2014 and updated AWGS in 2019. You may remove this information from method and result sections.
4. Line 190: Please write open form of the abbreviation CCI

Discussion

1. Line:246-248: ‘’Previous study from China in oncology surgical patients defined sarcopenia by the combination of low muscle mass and/ or low muscle strength and low physical performance’’. This information lacks the reference. You may add reference or may remove this information, the discussion is long anyway.
2. Dİscussion is too long, from lines 286 to 315 should be shortened
