# Supplementary material for: A simpler screening tool for sarcopenia in surgical patients
Source: PLoS One. 2021 Sep 23;16(9):e0257672. doi: 10.1371/journal.pone.0257672 (PMC8460047; doi:10.1371/journal.pone.0257672)
Supplement: Attachment — Submitted filename: Review Plus-One.docx [file pone.0257672.s003.docx]

Dear editor,

I read the article entitled “The validity of a simple screening tool for sarcopenia in surgical patients” with great interest. Screening and diagnosis of sarcopenia is important in geriatric assessment. Sarcopenia, the age-associated loss of skeletal muscle mass, has been postulated to be a major factor in the strength decline with aging. Moreover, sarcopenia is related to functional impairment, disability, falls, and loss of independence in older adults. In clinical use there are some screening tools for sarcopenia. Therefore, new tools may be tried for screening and / or diagnostic purposes. Accordingly, this study provides important data. A lot of efforts have been put together for this study. I congratulate the authors for the study because this is a worthwhile study and can be considered for publication. However, there are some major points that should be revised substantially.  Please find my comments below.

**Abstract**

1- In the background part its written that ‘’...muscle functions (strength and function)": this phrase is confusing? What do you indicate by function within the parenthesis?
2- ‘’ ….which is costly and immobilization’’ This phrase also makes no sense.
3- In line 27, We differentiate between "assessment" and "screening". Assessment is used for diagnosis whereas screening is for screening. That is to further select cases for assessment (diagnostic) protocols. Please express your intend in a correct way.
4- In line 28, What kind of an assessment tool is this? Is it something you suggest or already has been suggested in previous studies? If this was your first-time suggestion, then you should give details on its components.
5- In line 29, ‘’ ...diagnosis performance..’’ It should be "diagnostic performance" In general there are flaws in use of English. English should be edited by a native speaker preferably
6- In line 29, Instead of elderly, please use "older adults".
7- In the results part line 36, malnutrition and underweight are very inter-related. I do not think that they can be included as independent variables within the same regression analysis. Please clarify.
8- In line 39, ‘’….risk of malnutrition & malnutrition and/ or abnormal physical performance.’’ As far as I understood, it is better to write as “...risk of malnutrition/malnutrition". Also what do you mean by writing “...and/or .."
9- In conclusion part, line 43: We do not screen sarcopenia by muscle mass measurement. The authors might have had a confusion in this regard.
10- In the keywords, assessment tool is not the right word here. Consider screening tool.

**Background**

1- Two right square brackets in some references, please correct them.
2- In line 58 ‘’ Recent guidelines from the American College of Surgeons emphasize the importance of assessing sarcopenia prior to oncologic surgery in elderly patients.’’ In the referenced guideline they were not suggest assessing sarcopenia prior the surgery. They recommended document functional status, history of falls and frailty.
3- In line 62-65 ‘’ In essence….’’ This sentence is difficult to understand. Please describe better.
4- In line 69 ‘’… muscle quantity and quality’’ instead of "and", you should use "and/or".
5- In line 71-75 ‘’ Concerning the assessment of muscle mass….’’ Here, BIA comes forward due to its practical and portable application without exposure to any radiological harms. The authors are recommended to emphasize this fact, they may consider PMID: 28414253 to refer for this information.
6- In line 80 its written ‘assessment tool’. not assessment. Please be careful in your statements, particularly if your aim is to suggest a "screening" tool. Please review and correct your manuscript in terms of incorrect use of "assessment" word instead of screening.
7- In line 83-86 ‘’ Malnourished surgical……’’ After this sentence, the authors should develop the underlying the logic why they intended to use malnutrition as a component of sarcopenia screening in these patients.
8- Its written that the aims of this study were to design and validate the diagnosis performance of a simple assessment tool for screening sarcopenia in elderly cancer patients. There are some simple screening tools, such as SARC-F (PMID: 27066316 ). The authors should denote particularly SARC-F, as it is a very simple and convenient tool that demonstrated ability to predict adverse outcomes and sarcopenia, esp. the probable sarcopenia (PMID: 27066316 , PMID: 30272090). It has also potential other applications besides sarcopenia, such as frailty (PMID: 33786561). Moreover, SARC-F has been reported to screen for sarcopenia by application of alternative/lower cut-off scores (https://doi.org/10.1007/s12603-021-1617-3). SARC-F is also suggested by EWGSOP2 for "formal screening" and a project to widen its use has been endorsed by EuGMS (https://doi.org/10.1007/s41999-017-0003-5). Also, consider use of SARC-F by AWGS recommendation. These information on SARC-F should be noted in few sentences in order to avoid from a biased presentation of the literature. Moreover, I would suggest including at least a sentence on Ishii screening tool (PMID: 24450566). Also, SARc-CAlF which incorporates calf circumference as an indirect measure of muscle mass should be noted with few sentences (PMID: 27650212 and PMID: 30379299).

**Materials and Methods**

1- Its written that it is longitudinal and cross–sectional study. How can a study be both "longitudinal" and cross-sectional? It is conflicting.
2- In line 102 ‘Patients unable to walk or stand up were excluded.’ This makes that the proposed screening tool cannot be applied to those that are unable to walk, etc. This should be signified in the limitations section of the Discussion. Also, the authors should describe why they excluded these subjects.
3- In line 106 about the BIA measurement, Edema/ major fluid electrolyte abnormalities also precludes BIA assessment. Have you excluded those as well?
4- In line 118, the scoring categorization of MNA-SF should be integrated.
5- In line 119-120 measuring muscle mass with BIA, Is this appendicular muscle mass or total? You should specify.
6- In line 133-137 about the definition of sarcopenia, these are somewhat older references to define/diagnose sarcopenia. As authors would know, there are more updates diagnostic recommendations on sarcopenia both in Europe and Asia. This should be noted and discussed in the discussion section as a limitation of the study. Also, I guess the reference 27 should be corrected as reference 6.
7- In statistical analysis, have you checked normality? And how?

**Results**

1- In line 177 and 179 ‘Eleven patients..., five patients...’ Give % to allow readers understanding the impact of sarcopenia in decision processes better.
2- In line 190 ‘ …the sarcopenic group had a significantly lower number of patients with DM and DLP.’ This should be discussed with few sentences. Hypothetically sarcopenia should be related with higher DM and dyslipidemia due to loss of metabolic active muscle tissue. However, authors should clarify that sarcopenia is reported associated with low DM if muscle mass is adjusted by height2 but with higher DM if muscle is adjusted by weight/BMI (may refer <https://doi.org/10.1016/j.eurger.2015.12.012>).
3- In multivariate analysis, the authors should specify the dependent variable and independent variables in the regression analysis. This should also be given in the Table with a footnote.
4- In line 196, Barthel index is non-significant. Needless to state here.
5- About the formula 1, The authors should acknowledge that there is "probable sarcopenia" diagnosis that can be already made by solely measuring hand grip strength. The authors should specify that they are indicating confirmed sarcopenia or sarcopenia definitions that incorporate muscle mass.
6- Authors are recommended to give AUC values for the formulas as well.
7- In line 216, its written that EWGSOP2 demonstrated the highest specificity. Do you indicate confirmed sarcopenia or probable sarcopenia definitions of EWGSOP2 here?

**Discussion**

1- Discussion is too long. The authors should first outline main findings of their study and then discuss the findings by comparing with the similar studies in the literature.
2- In line 236 ‘In addition to the prevalence of sarcopenia being 34% , the prevalence rate increased with age...’ : I do not think that such detailed discussion on prevalence of sarcopenia in this study and comparison with others is needed. The objective of this study is NOT to report prevalence of sarcopenia. Your objective is to evaluate screening ability of the formulas you suggested. Therefore, shorten the Discussion and be sure that you discuss your findings around your main objective.
3- In line 263 ‘Regarding the factors related to sarcopenia, we found older age, malnutrition, and underweight status to be significantly associated with sarcopenia.’ : Refer to my previous comment on sarcopenia prevalence of sarcopenia/discussion. Concentrate on your main findings, not the secondary outputs. Just few sentences instead of this huge paragraph would be enough.
4- In line 295 they mentioned about SARC-F. My recommendations to note on SARC-F and Ishii tool in the Introduction sections may be detailed and answered at this part of the Discussion section. Nevertheless, some introductory sentences on SARC-f and Ishii should be stated in the Introduction section as well.
