# Supplementary material for: A simpler screening tool for sarcopenia in surgical patients
Source: PLoS One. 2021 Sep 23;16(9):e0257672. doi: 10.1371/journal.pone.0257672 (PMC8460047; doi:10.1371/journal.pone.0257672)
Supplement: Attachment — Submitted filename: Review PLOSOne-The validity of a simple screening tool.docx [file pone.0257672.s005.docx]

Dear Editor,

I previously made my recommendations for revision of the paper titled **‘The validity of a simple screening tool for sarcopenia in surgical patients’**. However there still seems some flaws in the writing that should be fixed properly. Below you can find my new recommendations.

*English should be improved in the whole writing.

**Abstract**

***Page 2, Line 27:** ‘Non-portable’ should be used instead of immobile device.

***Page 2, Line 28:** ***The aim of this study was to design and validate the diagnostic performance of a simple screening tool for screening sarcopenia***

These formula are not suggested for diagnosis of sarcopenia. Therefore, ‘diagnostic’ term should not be used.

***Page 2, Line 32:** The details about this screening tool should be explained in methods part. Which parameters were considered for analyses, the details about formula should be briefly mentioned.

***Page 2, Line 37:** Malnutrition and underweight status are surely expected to be strongly related. Therefore, before putting into the same regression analysis, multicollinearity should be checked.

**Background**

***Page 3, Line 57:** In whole writing, elderly term should be changed to older adults.

***Page 3, Line 62:** ***Muscle function can be divided into those that require both muscle strength and physical functionality or only one of these elements.***

Functionality should be revised as ‘performance’.

*Introduction should be shortened.

**Materials and methods**

***Page 5, Study population exclusion criteria:** Edema can also affect the BIA results, therefore, measurements without regarding edema status should also be stated as a limitation of the study.

***Page 7, Line 155: *At risk and malnutrition and underweight BMI were considered to be factors for diagnosing sarcopenia.***

Underweight BMI should be changed as ‘Being underweight by BMI’. Again, language revision should be done seriously.

**Results**

***Page 12, Line 249:** ***Diagnosis performance a simple tool for screening sarcopenia***

Diagnostic performance term should not be used. These formula were suggested for sarcopenia screening, so 'screening' and 'diagnosis' terms should not be used interchangeably.

***Page 13, Line 256: *EWGSOP2 demonstrated the highest specificity (100%).***

Whether the high specificity proposed by EWGSOP2 is for probable sarcopenia or confirmed sarcopenia should be stated.

**Discussion**

***Page 18, Line 355: *Although, there was a slightly low specificity and PPV in female, we introduced a practical algorithm for a diagnosis of sarcopenia in elderly cancerous surgical patients without using muscle mass measurement (Fig 2).***

The algorhythm the authors proposed for sarcopenia diagnosis seems problematic. The authors should decide whether they propose this formula for screening (finding cases) or assessment of sarcopenia. In addition, muscle mass measurement was included for diagnosis in the algorhythm. But they claimed that the algorhythm enables to diagnose sarcopenia without muscle mass measurement. There seems a paradox.

**Conclusion**

*Conclusion should be shortened. The first two sentences are the findings previously mentioned, unnecessary to repeat at the end.
