# Supplementary material for: A simpler screening tool for sarcopenia in surgical patients
Source: PLoS One. 2021 Sep 23;16(9):e0257672. doi: 10.1371/journal.pone.0257672 (PMC8460047; doi:10.1371/journal.pone.0257672)
Supplement: Attachment — Submitted filename: second review.docx [file pone.0257672.s006.docx]

Most of my concerns are addressed, but the text still needs a minör revision:

1.Please prefer the term “older adults” instead of “elderly” throughout the text

2.The text still needs a rigorous editing,

3.My suggestions for some phrases that cause misunderstanding are as follows:

Introduction

Line 77-78: The sentence’’ the DXA and BIA have some limitation in terms of the accessibility, and costly equipment’’. Please add the plural suffix ‘’s’’ to the word limitation. Instead of ‘’costly equipman’’ please write ‘’cost’’ only.

Line 95: In the sentence …….any one tool……, please remove the word ‘’one’’, write as ….any tool…

Line 97: Instead of ‘’diagnosis performance’’, please write ‘’diagnostic performance’’

Material and methods

Line 128: Other collected data included……Here the term other refers to components of MNA-SF tool. But it is perceived as a different data apart from MNA-SF tool. I recommend combine with previous sentence and you may write as ‘’ Preoperative nutritional screening was performed using the Mini Nutritional Assessment - Short Form (MNA® 128 –SF), which included reduction in dietary intake within the past three months, body mass index (BMI), history of weight loss within the last three months, mobility, psychological stress and/ or acute disease within the past 3 months, and neuropsychological problems.’’

Results

Line 249: Please rewrite subheading, you may write as ‘’ Diagnostic performance of a simple tool for screening sarcopenia’’

Line 250-252: Please rewrite the sentence ‘’The combination of low muscle strength and risk of malnutrition & malnutrition and/ or abnormal physical performance (C3) showed the highest sensitivity and accuracy either the AWGS or the updated AWGS as the gold standards’’you may write as ‘’ The combination of low muscle strength and risk of malnutrition & malnutrition and/ or abnormal physical performance (C3) showed the highest sensitivity and accuracy when using the AWGS or the updated AWGS as the gold standards’’

Line 252-254:Please rewrite the sentence ‘’ The sensitivity, specificity, accuracy and AUROC were 81.0%, 78.4%, 79.3% and 0.8, respectively as compared to C1, C2 and C4 when using AWGS as a gold standard’’. In this sentence percent results are belong to C3, but C3 is not mentioned in the sentence, in addition sentence gives the statistical information only related to C3, there is not a comparision between the statistical results of the formulas. You may rewrite the sentence as ‘’ The sensitivity, specificity, accuracy and AUROC of C3 were 81.0%, 78.4%, 79.3% and 0.8, respectively when using AWGS as gold standard.’’

Line 264-266: with similar reasons as above mentioned you may rewrite the sentence as ‘’ The sensitivity, specificity, accuracy, and AUROC of C3 were 80% , 68. 5% , 73. 3% and 0.74 respectively when using the updated AWGS criteria as gold standard (Table 4b).’’

Discussion

Line 306-308: Please rewrite the sentence, you may rewrite as ‘’ The combination of low muscle strength and risk of malnutrition & malnutrition and/or abnormal physical performance (C3), demonstrated high sensitivity, specificity, and predictive power when validated against a consensus of the Asian Working Group for Sarcopenia (AWGS).

Line 329: Please write immobile instead of immobilization
